# Biocalcifying Potential of Ureolytic Bacteria Isolated from Soil for Biocementation and Material Crack Repair

**DOI:** 10.3390/microorganisms10050963

**Published:** 2022-05-03

**Authors:** Laxmi Leeprasert, Duenrut Chonudomkul, Chanita Boonmak

**Affiliations:** 1 Department of Microbiology, Faculty of Science, Kasetsart University, Bangkok 10900, Thailand; mochii.rey@gmail.com (L.L.); fscidrc@ku.ac.th (D.C.); 2 Biodiversity Center Kasetsart University (BDCKU), Bangkok 10900, Thailand

**Keywords:** ureolytic bacteria, calcite, MICP, urease

## Abstract

Microbially induced calcium carbonate precipitation (MICP) has been highlighted for its application in civil engineering, and in the environmental and geotechnical fields. Ureolytic activity is one of the most promising bacterial mechanisms in terms of inducing calcium carbonate formation. In this study, four bacterial isolates with high-yield urease production capabilities were obtained from two-step screening using a high-buffered urea medium. The highest urease activity and calcium carbonate formation was observed in *Lysinibacillus fusiformis* 5.1 with 4.40 × 10^3^ unit/L of urease and 24.15 mg/mL of calcium carbonate, followed by *Lysinibacillus xylanilyticus* 4.3 with 3.93 × 10^3^ unit/L of urease and 22.85 mg/mL of calcium carbonate. The microstructure of the precipitated crystalline calcium carbonate was observed using scanning electron microscopy. X-ray diffraction analysis confirmed that the main polymorph of the calcium carbonate particle obtained from both isolates was calcite. Examination of the material-crack filling in mortar specimens showed that calcite layers had formed along the crack edges and inside after 10 days, and gradually filled the cracks up to the upper surface. These results showed that these two isolates presented robust characteristics of potential MICP-inducing bacteria for civil engineering and material engineering applications.

## 1. Introduction

Microbially induced calcium carbonate (CaCO_3_) precipitation (MICP) is a biomineralization process that naturally occurs in an environment, with CaCO_3_ being formed as a result of microbial metabolic activity. MICP can be performed through various microorganism mechanisms such as urea hydrolysis, photosynthesis, sulfate reduction, nitrate reduction, or other biochemical activities of the microorganisms that increase the saturation state of carbonate [1,2,3,4]. The biological process has advantages over the chemical method in terms of economic and environmentally friendly aspects. Current trends in MICP technology focus on ground improvement, crack-sealing, self-healing concrete and the removal of heavy metals from water. The bacteria used in the process are mostly encountered in harsh environments; mainly of high alkalinity, nutrient deficiency and high shearing force. The high viability and enzyme activity of the bacteria are essential factors in the success of the MICP process [1,5,6,7]. In some applications such as self-healing concrete when bacteria are incorporated into concrete during the mixing and casting process, using spores of alkali-tolerant bacteria is more favorable than using vegetative cells because they can resist harsh environments [8,9]. Ureolytic bacteria, urease-producing bacteria, are one of the most widely-studied MICP-inducing microorganisms. The urease enzyme hydrolyzes urea to produce ammonia and carbonic acid. Ammonia is equilibrated in water to form ammonium and hydroxide ions. The hydroxide ions result in an increase in pH, which shifts the carbonic acid equilibrium, resulting in the formation of carbonate. Meanwhile, the anionic cell components of bacteria, such as extracellular polymeric substances (EPS) and cell walls, provide nucleation sites that attract the surrounding metal ions, such as the Ca^2+^ ion, to bind with carbonate ions and precipitate as CaCO_3_ [5,7,10,11,12,13]. Applications of MICP in actual environments can be achieved through bio-stimulation [14,15] and bio-augmentation using indigenous bacteria (strains obtained from the native site) [16,17,18] or exogenous bacteria (non-native bacterial strains) [9,15,19,20,21,22]. The efficiency of the on-site MICP treatment mainly depends on bacterial metabolism and the interaction between the ureolytic bacteria, indigenous microorganisms, and abiotic factors in the environment. Hence, the selection of suitable MICP-inducing strains is one of the key processes for practical implementations of the MICP technique. Most research on ureolytic-based MICP have been focused on *Sporosarcina pasteurii* strains due to their high ureolytic ability [5,15,19,20,21,22,23]. Therefore, it is important to isolate and investigate other potential urease-producing bacteria such as *Bacillus* [2,4,23], *Lysinibacillus* [9,17,24,25], *Pararhodobactor* [16] and *Psychrobacillus* [18] to provide bioresources which can be utilized in various climates and environments.

Recently, the application of MICP in civil engineering has been widely studied, including in Thailand [9,19,26]. However, a pure culture of ureolytic bacteria originally isolated in Thailand has not yet been obtained and characterized. This study aimed to isolate and screen for high-yield urease-producing bacteria with the ability to precipitate large amounts of CaCO_3_. The produced CaCO_3_ crystals were characterized. The efficiency of crack filling in mortar samples and the storage testing of bacterial endospores were investigated. Moreover, a shelf-life prediction for commercial production of the MICP-inducing bacteria was also evaluated. From our knowledge, this is the first report of shelf-life storage of the MICP-inducing bacteria using accelerated storage testing.

## 2. Materials and Methods

### 2.1. Sampling Site and Sample Collection

In November 2017, a total number of seven soil samples were collected. Six samples were collected from different areas of two mangrove forests in Thailand: the Phra Chedi Klang Nam Mangrove Forest Learning Center, Rayong province (*n* = 3), and the Black Sand Beach and Mangrove Forest Natural Tourism Center, Trat province (*n* = 3). In addition, one sample of sandy loam was obtained from an area with ground subsidence containing a factory in Bowin, Si Racha district, Chon Buri province. From each sampling site, a soil sample (approximately 500 g) was taken from a depth of 1–5 cm and brought to the laboratory in an ice box. It was then kept at 4 °C until use for bacterial isolation.

### 2.2. Isolation and Screening for High-Yield Urease Producing Bacteria

Isolation of the ureolytic bacteria was performed using dilution plate and direct streak methods. For dilution plating, one gram of soil sample was suspended and diluted to 10^−1^ to 10^−5^ by 0.85% saline solution, then 0.1 mL of each dilution was spread in duplicate on the surface of Christensen’s urea agar (1 g/L peptone, 5 g/L NaCl, 1.2 g/L Na_2_HPO_4_, 0.8 g/L KH_2_PO_4_, 1 g/L glucose, 20 g/L urea, 0.012 g/L phenol red, 15 g/L agar, pH 6.8–7.0). A soil suspension with a dilution of 10^−1^ was directly streaked onto three plates of Christensen’s urea agar. The plates were incubated at room temperature (28 ± 2 °C) for 24–48 h. Distinct colonies with a bright fuchsia color appearance around the colonies indicating urease activity were selected and purified on nutrient agar (3 g/L beef extract, 5 g/L peptone, 15 g/L agar, pH 6.8–7.0). The screening of high-yield urease-producing bacteria was performed using urea medium with a high buffering capacity. An overnight culture of selected isolates on nutrient agar was suspended in 0.85% saline solution to a turbidity of 4.0 McFarland standard, and used as an inoculum. One hundred and fifty microliters of bacterial suspension were added to 5 mL of Stuart’s urea broth (0.1 g/L yeast extract, 9.1 g/L KH_2_PO_4_, 9.5 g/L K_2_HPO_4_, 20 g/L urea, 0.012 g/L phenol red, pH 6.8–7.0), incubated on a rotating shaker at 150 rpm at room temperature for 48 h. *Escherichia coli* was used as a negative control. The color change of the phenol red was observed at 6, 8, 10, 12, 18, 24, 36 and 48 h.

### 2.3. Bacterial Identification

The 16S rRNA gene of the selected ureolytic bacteria was amplified from genomic DNA using the universal bacterial primers 27F (5′-AGAGTTTGATCMTGGCTCAG-3′) and 1492R (5′-TACGGYTACCTTGTTACGACTT-3′). Polymerase chain reaction (PCR) was performed with a Bio-Rad T100 Thermal Cycler (Bio-Rad Laboratories, Hercules, California, USA) using the following cycling conditions: 94 °C for 90 s, followed by 25 cycles of 94 °C for 30 s, 55 °C for 30 s, and 72 °C for 1 min, and then a final extension step at 72 °C for 5 min. The nucleotide sequences were compared to sequences of type strains in the EzBioCloud database [27] for taxonomic identification. A phylogenetic tree was constructed from the evolutionary distance data using the Neighbor-Joining method [28] based on the Maximum Composite Likelihood model [29] of MEGA11 software [30]. Confidences for the phylogenetic tree were estimated from bootstrap analysis (1000 replicates) [31]. The 16S rRNA gene sequences of the isolates 2.2, 3.7, 4.3 and 5.1 were submitted to the GenBank database under accession number LC672026-LC672029, respectively. 

### 2.4. Determination of Bacterial Growth and Urease Assays

Seed cultures were grown on nutrient agar or tryptic soy agar (TSA; Himedia, India) for 16–18 h. To prepare the inoculum, the bacterial culture was suspended in a 0.85% saline solution to achieve a turbidity of 0.5 McFarland standard (approx. OD_600_ = 0.1). Bacterial growth was examined in four media including nutrient broth (NB), NB supplemented with 2% of urea, tryptic soy broth (TSB) and TSB supplemented with 2% of urea. The total volume of 6.25 mL of inoculum (5% *v*/*v* of medium) was transferred to 500-mL Erlenmeyer flasks containing 125 mL of the test medium. The cultures were incubated on a rotary shaker at 150 rpm and 28 °C for 48 h. The culture broth was collected at 0, 2, 4, 6, 8, 10, 12, 18, 24, 30, 36, 42, and 48 h. The optical density of the cells was measured at 600 nm using a spectrophotometer (Shimadzu, Kyoto, Japan). The urease activity at 8, 12, 24 and 48 h of incubation was examined by using a urease activity assay kit MAK120 (Sigma-Aldrich, St. Louis, MO, USA).

### 2.5. Determination of EPS Formation

Seed cultures of the selected ureolytic bacteria were grown on TSA for 16–18 h. The culture broth was suspended in fresh TSB. The initial cell optical density was adjusted to an OD_600_ value of 0.2 (approx. 3 × 10^8^ CFU/mL). A total volume of 200 µL of each diluted culture was transferred to separate wells in a 96-well plate and incubated at 25 °C for 24 and 48 h. EPS quantification was determined using a biofilm formation assay previously described by [32]. After incubation, the culture broth and the planktonic cells were removed and rinsed with distilled water. The EPS attached to the well was stained by 100 μL of 0.1% *w*/*v* crystal violet for 10 min. Each well was washed with distilled water twice to remove excess crystal violet. The adherent crystal violet was eluted by equal amounts of 33% acetic acid for 10 min and observed by measuring at an absorbance of 595 nm with a microplate reader. Sterile medium was used as a blank control.

### 2.6. Determination of Calcium Precipitation Activity

A total volume of 6.25 mL of the active culture of the ureolytic bacteria with 0.2 value of OD_600_ was transferred to 500-mL Erlenmeyer flasks containing 125 mL of TSB supplemented with 2% of urea, and incubated on a rotary shaker at 150 rpm, at 28 °C for 48 h. To investigate the influence of the bacterial cells as a nucleation site for CaCO_3_ precipitation, 50 mL of the culture broth, with and without cells, was used for the determination of the calcium precipitation activity. For preparation of the culture broth without cells, the bacterial cells were removed by centrifuging at 9000 rpm for 10 min. A CaCl_2_ solution was added to the supernatant to a final concentration of 0.1 M and 0.4 M CaCl_2_. The CaCO_3_ precipitate was collected, carefully washed with water, and the dry weight was measured after being kept at 100 °C until constant weight was achieved. All experiments were performed in triplicate. The CaCO_3_ particles were visualized by scanning electron microscopy (SEM) using an FEI Quanta 450 FEG scanning electron microscope (FEI Inc., Hillsboro, OR, USA) with 20 kV accelerating voltages. Crystalline property was determined by X-ray diffraction (XRD) analysis using a Bragg-Brentano D8 Advance diffractometer (Bruker, Billerica, MA, USA).

### 2.7. Material-Crack Filling on Sample Mortar

Examination of material-crack filling was performed using mortar specimens. Each mortar specimen was prepared in a 2.5 × 2.5 × 2.5 cm^3^ cube mold with a plastic sheet inserted to create a simulated crack (0.01–0.03 mm width and 0.5 mm depth). The simulated crack was confirmed to be of the appropriate size under a stereomicroscope. The mortar specimens were sterilized at 121.5 °C for 15 min, dried in a hot air oven at 100 °C, and kept in separate sterilized boxes. An EPS-producing ureolytic bacteria, *L. xylanilyticus* 4.3, and a non-EPS producing ureolytic bacteria, *L. fusiformis* 5.1, were grown in TSB supplemented with 2% of urea and incubated on a rotary shaker at 150 rpm and room temperature for 24 h. The cell pellets were centrifuged at 9000 rpm for 10 min and resuspended in fresh TSB supplemented with 2% urea. Cell density was adjusted to achieve an OD_600_ value of 0.5. The crack was filled with 100 µL of the cell suspension and 8 µL of 5 M CaCl_2_ (final concentration of 0.4 M CaCl_2_) every 12 h for 30 days, incubated at room temperature. After the material-crack filling process, the mortar specimens were dried in a hot air oven at 55 °C for 1 day and sputter coated with Au. CaCO_3_ crystallization was observed by SEM, and the mineralogy was determined by energy dispersive spectrometer (EDS) analysis. The SEM and EDS analyses were performed on a FEI Quanta 450 FEG scanning electron microscope (FEI Inc., USA) with 20 kV accelerating voltages.

### 2.8. Accelerated Storage Testing of Freeze-Dried Bacterial Endospores

*L. fusiformis* 5.1 and *L. xylanilyticus* 4.3 were grown in 500 mL Erlenmeyer flasks containing 125 mL of a modified sporulation medium [33]: 1 g/L beef extract, 5 g/L peptone, 2 g/L yeast extract, 0.25 g/L MgSO_4_, 1 g/L KCl, 0.15 mg/L FeSO_4_, 0.16 g/L Ca (NO_3_)_2_ and 1.26 mg/L MnCl_2._ The culture was incubated on a rotary shaker at 150 rpm at room temperature until bacterial spores were present and covered 90% of microscopic fields under a light microscope. The results of sporulation were examined using Schaeffer-Fulton‘s method every 0, 3, 5, 7, and 15 days. To kill the vegetative cells, the culture broth was treated with a heat shock process at 80 °C for 10 min and immediately chilled in an ice bath for 5 min. The pellets were harvested by centrifugation at 9000 rpm for 10 min and mixed with 10% skimmed milk in equal amounts. The samples were divided into 500 µL per Eppendorf tubes and frozen at −20 °C. A freeze-drying process was performed using a freeze dryer. The freeze-dried samples were stored at 8, 15, 37, and 55 °C. Viability assays were determined by a total viable count on TSA at 0, 7, 14, 21, 30, and 45 days. The slope of log viability versus time regression was used to obtain the specific rate of degradation per day (*k*) at each temperature, following Equation (1) [34].

Log*N* = log *N*_0_ − *kt* (1)

*N*_0_ is the initial number of viable cells, *N* is the number of viable cells at any time (both represented as CFU/mL), and *t* is the storage time in days.

*k* = *k*_0_e^(−*E*a/*RT*)^ (2)

The Arrhenius equation, as shown in Equation (2), can be used to evaluate the correlation between temperature (Kelvin) and *k* values. Where *k*_0_ is the pre-exponential constant, *E*_a_ is the activation energy for the reaction in kilojoules per mol, *R* is the universal gas constant (8.32 j/mol·K), and *T* is the absolute temperature in Kelvin. Equation (2) was used to obtain the predicted shelf life by calculating the predicted values of the specific rate of survival (*k* per day) at the different storage temperatures. 

### 2.9. Statistical Analysis

Statistical significance was evaluated using the SPSS statistics software with Duncan’s multiple range test of variance (ANOVA). The significance level of *p* < 0.05 was defined as a significant difference.

## 3. Results

### 3.1. Bacterial Isolation and Identification

**Figure 1:**
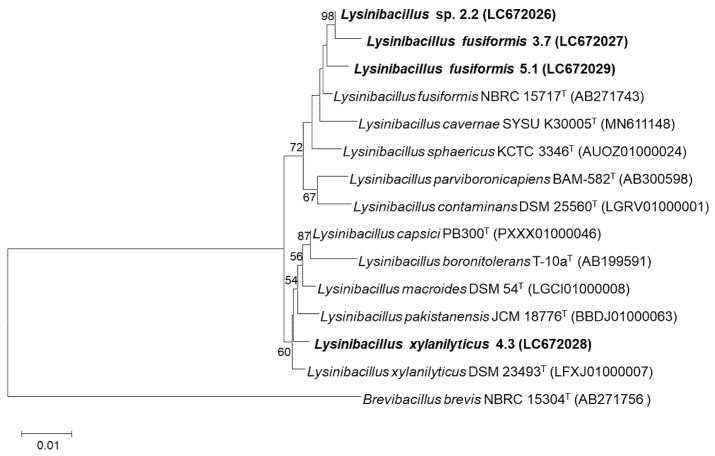
Phylogenetic placement of the isolated ureolytic bacteria and their closely related taxa based on sequence analysis of the 16S rRNA gene using the Neighbor-Joining method based on the Maximum Composite Likelihood model. Named in bold type are the isolates obtained from this study. Numbers on branches indicate percentages of bootstrap sampling (>50%), derived from 1000 samples. Bars, 0.01 substitutions per nucleotide position.

In the primary screening, ten isolates of ureolytic bacteria were obtained from seven soil samples on Christensen’s urea agar. However, only four isolates, isolate 2.2, 3.7, 4.3 and 5.1, were able to change the pH of the Stuart’s urea broth from an initial pH 7.0 to a pH of 9.82–10 within 72 h, and exhibited high-yield ureolytic ability (Table 1). The four isolates were Gram-positive, long rod shaped, swollen endospore-forming, catalase, and oxidase positive. Analysis of the16S rRNA gene compared with published sequences available in the EzTaxon database revealed that the four isolates belonged to the genus *Lysinibacillus*. The 16S rRNA sequence similarity threshold for the cut-off value at species level was 98.7% [35]. Therefore, isolate 4.3 was identified as *Lysinibacillus xylanilyticus*, with a 99.26% similarity to *Lysinibacillus xylanilyticus* 23493^T^, while the other two isolates, 3.7 and 5.1, were *Lysinibacillus fusiformis* with 99.32 and 99.42% similarity to *Lysinibacillus fusiformis* NBRC 15717^T^, respectively. Isolate 2.2 showed 98.35% similarity with its closest species, *L. fusiformis* NBRC 15717^T^, hence is represented as a possible novel bacterial species that requires further study. The phylogenetic placement of the four isolates within the genus *Lysinibacillus* is shown in Figure 1. 

### 3.2. Bacterial Growth, Urease Activity and EPS Formation

**Figure 2:**
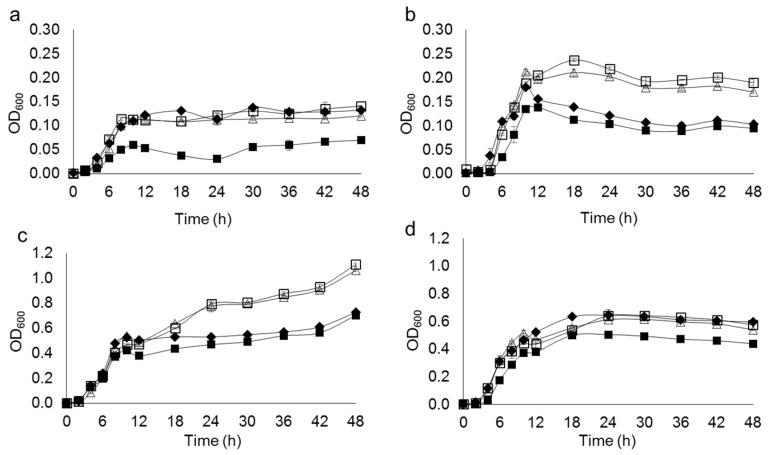
Growth of bacterial isolates in NB (**a**), NB supplemented with 2% urea (**b**), TSB (**c**), and TSB supplemented with 2% urea (**d**); *Lysinibacillus* sp. 2.2 (△), *L*. *fusiformis* 3.7 (□), *L. xylanilyticus* 4.3 (♦), *L. fusiformis* 5.1 (■). Values are mean ± SD. The SD value was shown in supplementary Table S1.

**Figure 3:**
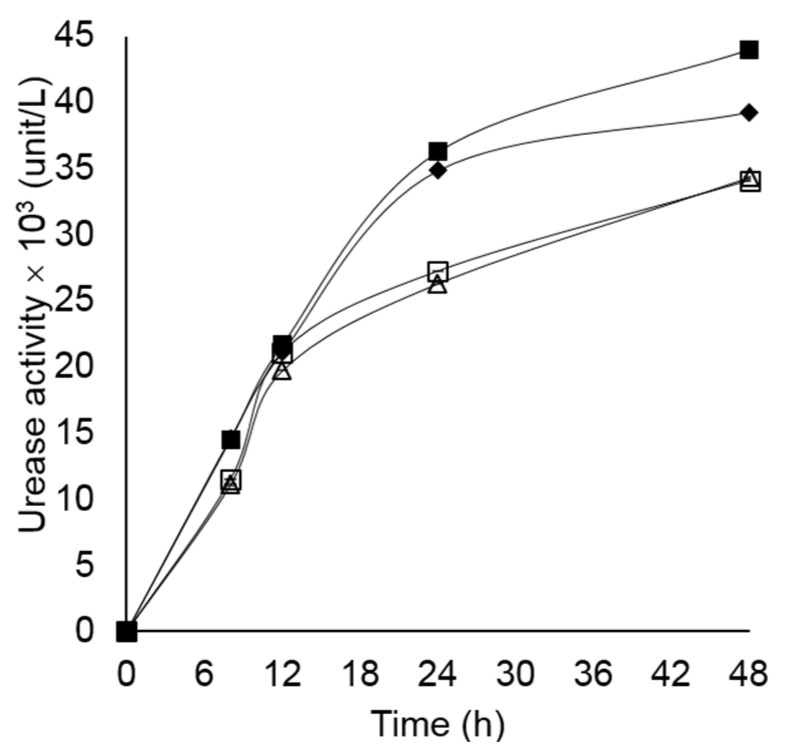
Urease activity of *Lysinibacillus* sp. 2.2 (△), *L*. *fusiformis* 3.7 (□), *L. xylanilyticus* 4.3 (♦), and *L. fusiformis* 5.1 (■) grown on TSB supplemented with 2% urea at 150 rpm, 28 °C for 48 h. Values are mean ± SD. The SD value was shown in supplementary Table S2.

**Figure 4:**
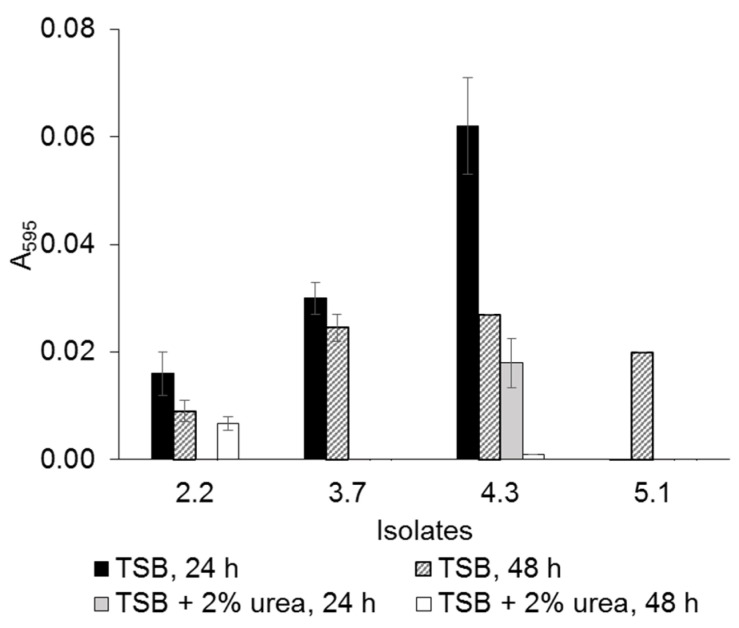
EPS formation in TSB and TSB supplemented with 2% urea at 25 °C for 24 and 48 h. Values are mean ± SD.

Bacterial growth in four different mediums is shown in Figure 2. The highest growth of all *Lysinibacillus* isolates was obtained in TSB, indicating that it was a more suitable medium for the bacterial growth than NB. In the NB, which contained a lower amount of nutrients compared to TSB, urea played a role as a supplemental nutrient. Therefore, the isolates grew better in the presence of urea. However, in the TSB, which was more enriched, the bacterial biomass reached 3–8 fold higher than the growth in NB within 12 h. Hence, a large amount of enzyme urease, which is a primary metabolite of ureolytic bacteria, was produced in the presence of urea, leading to a dramatical increase in pH value in the culture. This eventually inhibited the bacterial growth after 18 h of incubation. From the preliminary results, TSB was selected as the base medium for further experiments. The highest amount of urease was observed in *L. fusiformis* 5.1 (4.40 × 10^3^ unit/L) followed by *L. xylanilyticus* 4.3 (3.93 × 10^3^ unit/L), grown in TSB supplemented with 2% urea at 150 rpm, 28 °C for 48 h (Figure 3). The presence of urease at 6-h of incubation time indicated that the isolates had produced urease as a primary metabolite. The anionic charge of EPS provides a nucleation site of CaCO_3_ crystals by attracting calcium ion cation which facilitates precipitation. All isolates were able to produce EPS in TSB (Figure 4). However, the EPS formation was dramatically decreased in the presence of urea. Only two isolates, *Lysinibacillus* sp. 2.2 and *L. xylanilyticus* 4.3. produced EPS in TSB supplemented with 2% urea, with *L. xylanilyticus* 4.3 producing the greatest amounts of EPS at 24 h of incubation.

### 3.3. CaCO_3_ Precipitation and Characterization

**Figure 5:**
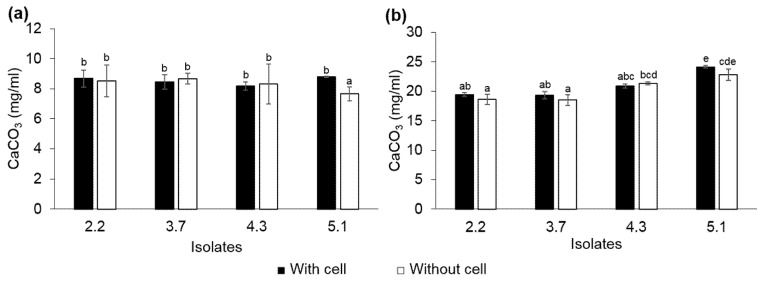
Precipitation of CaCO_3_ by the ureolytic bacteria grown in TSB supplemented with 2% urea, incubated for 48 h with addition of 0.1 M (**a**) and 0.4 M (**b**) CaCl_2_. Values are mean ± SD. Different letters indicate significance differences (one-way ANOVA; *p* < 0.05; Duncan’s multiple range test).

**Figure 6:**
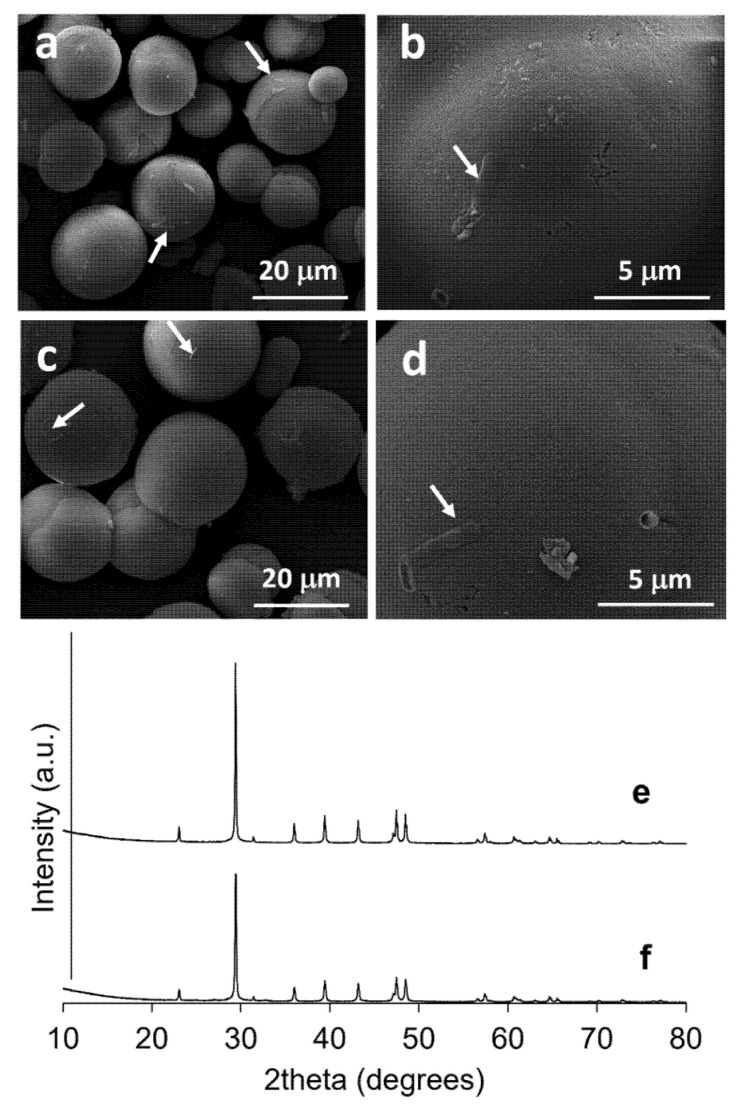
SEM images and XRD spectra of CaCO_3_ particles precipitated in the culture broth of *L. fusiformis* 5.1 (**a**,**b**,**e**) and *L. xylanilyticus* 4.3 (**c**,**d**,**f**) after addition of 0.4 M CaCl_2_. Arrow shows bacterial cells.

In this study, ureolytic bacteria were grown in TSB supplemented with 2% urea for 48 h before being precipitated with CaCl_2_ to avoid bacterial growth inhibition from excessive amount of calcium ion [36]. The CaCO_3_ precipitant was immediately observed after adding the CaCl_2_ solution, as a result of a chemical reaction. With the addition of 0.1 M CaCl_2_, the CaCO_3_ precipitant of cultures with and without bacterial cells weighed between 8.20–8.79 and 7.66–8.66 mg/mL, respectively. Meanwhile, in the presence of 0.4 M CaCl_2_, the CaCO_3_ precipitant of cultures with and without bacterial cells weighed between 19.34–24.15 and 18.52–22.85 mg/mL, respectively. After CaCO_3_ precipitation, the alkaline pH of the culture broth was immediately decreased from 9.25–9.33 to 8.13–8.31, i.e., close to neutral. The results showed that the concentration of calcium ions in the environments greatly affected the efficiency of the CaCO_3_ precipitation (Figure 5). Without calcium ion interference with regard to the bacterial growth and metabolism, urease of the ureolytic bacteria was a produced, degraded urea, and this created the excess amount of carbonate in the culture broth. Thus, *L. fusiformis* 5.1 and *L. xylanilyticus* 4.3 could precipitate CaCO_3_ to a slightly greater extent than the other isolates because they were able to produce a greater amount of urease (Figure 4 and Figure 5b). The dry weights of the CaCO_3_ particles in the presence and absence of bacterial cells were not significantly different (Figure 5). Therefore, the concentration of calcium ions and carbonate in the culture broth was probably the main factor, rather than the presence of nucleation sites in this condition. However, several bacterial imprints were observed on the surface of the CaCO_3_ particles (Figure 6), indicating that bacterial cells still served as nucleation sites, which agrees with previous reports (2, 21, 23). From these results, the highest urease producing isolate, *L. fusiformis* 5.1, which was not able to produce EPS in the presence of urea, and the second-best urease producing isolate with potential EPS ability, *L. xylanilyticus* 4.3, was selected to characterize CaCO_3_ crystals and be used in further experiments.

The morphology of the CaCO_3_ particles produced in the culture broth of *L. fusiformis* 5.1 and *L. xylanilyticus* 4.3 was observed under SEM, as shown in Figure 6. Irregular spherical particles with sizes ranging between 9.68 and 22.90 μm were mainly observed. A small number of smaller rod-shaped particles was detected. Bacterial cells were also found on the surface of the CaCO_3_ particles. The XRD spectra were used to analyze the mineralogy of the precipitant using a CuKα radiation source (λ = 1.5406 Å). The XRD patterns which overall ranged from 10° to 80° (2θ), confirmed that the CaCO_3_ polymorph of the particle from both isolates was calcite (Figure 6e,f). 

### 3.4. Material Crack Filling and Crystallization 

**Figure 7:**
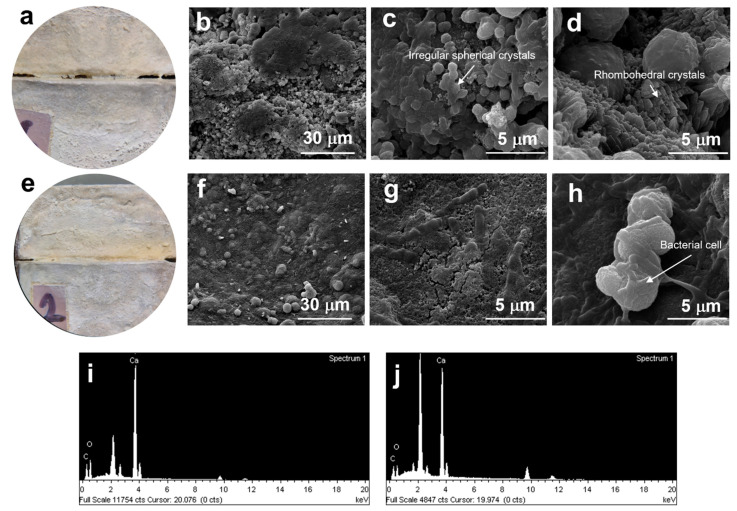
Microscopy images of the filled-cracks, SEM images and EDS spectra of CaCO_3_ particles in the mortar specimens induced by *L. fusiformis* 5.1 (**a**–**d**,**i**) and *L. xylanilyticus* 4.3 (**e**–**h**,**j**).

An examination of material crack filling was performed in mortar specimens with 0.01–0.03 mm wide simulated cracks using *L. fusiformis* 5.1 and *L. xylanilyticus* 4.3. White to yellow crystalline layers were clearly observed at the crack edges and inside the crack after 10 days and gradually filled up to the upper surface of the crack. The microstructure of the mortar specimens was examined using SEM as shown in Figure 7. Bacterial cells were observed on the surface of the crystalline layers inside the crack at 1500×. The crystal morphology of *L. fusiformis* 5.1 appeared to be irregular and spherical shaped crystals. However, the crystalline layer of *L. xylanilyticus* 4.3 appeared to form a smooth surface (Figure 7b,f). The EDS spectra confirmed that the crystals and the smooth layer were mainly composed of calcium, carbon, and oxygen, with a weight ratio that approximately matches CaCO_3_ (Figure 7i,j).

### 3.5. Accelerated Storage Test of Bacterial Endospores

**Figure 8:**
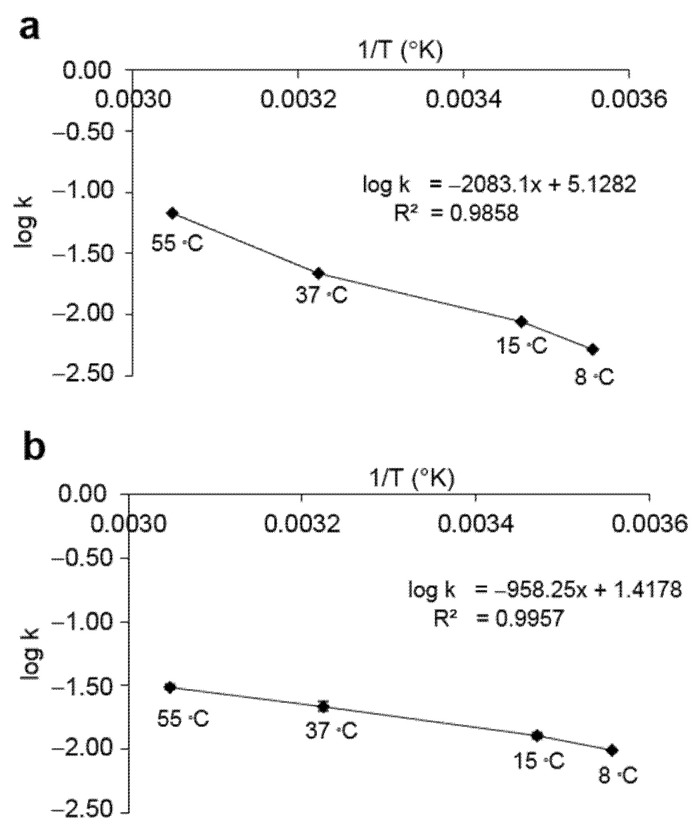
Arrhenius plots of the logarithms of the specific rates thermal degradation of the freeze-dried spores of *L. fusiformis* 5.1 (**a**) and *L. xylanilyticus* (**b**) after storage at 8, 15, 37 and 55 °C for 30 days.

**Figure 9:**
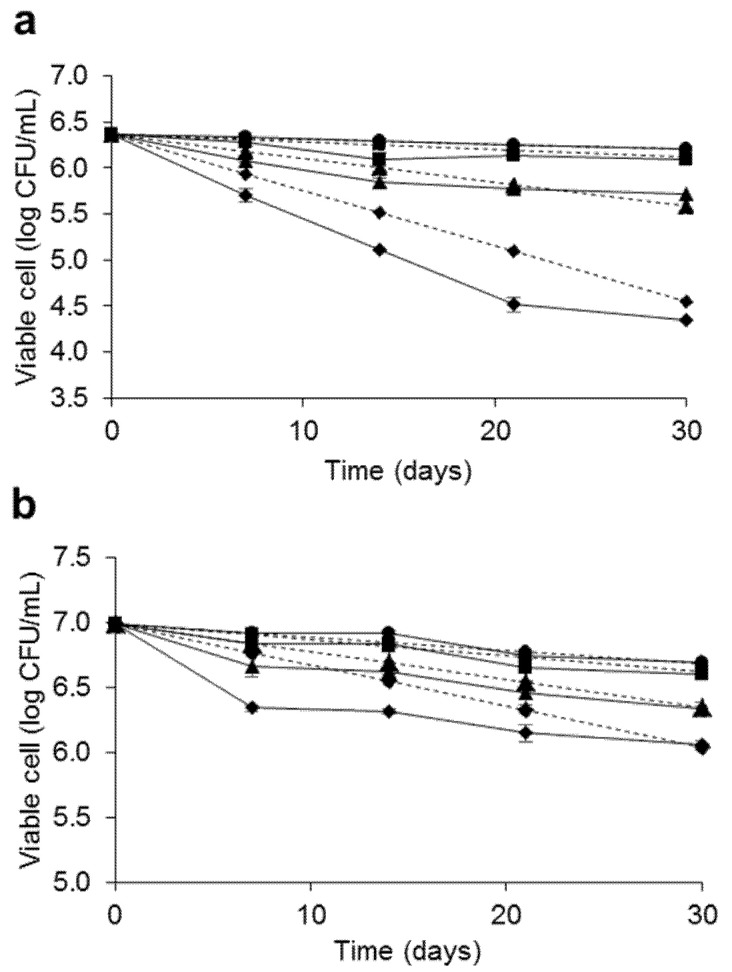
Accelerated stability of the freeze-dried spores of *L. fusiformis* 5.1 (**a**) and *L. xylanilyticus* 4.3 (**b**) at 8 (●), 15 (■), 37 (▲) and 55 °C (♦); –––, experimental and––, predicted viability. Values are mean ± SD. The SD value was shown in supplementary Table S3.

To evaluate the resistance of the bacterial endospores in terms of long-term preservation, accelerated storage testing of freeze-dried bacterial endospores was performed in aerobic conditions. The initial number of spores after the freeze-drying of *L. fusiformis* 5.1 and *L. xylanilyticus* 4.3 was 2.17 × 10^6^ CFU/mL and 1.23 × 10^7^ CFU/mL, respectively. The logarithms of the experimental specific rate of degradation per day (*k*) was plotted against storage temperatures of 8–55 °C, as shown in Figure 8. The effect of storage temperature on spore viability indicated that the spores of *L. fusiformis* 5.1 were more resistant to high storage temperatures than *L. xylanilyticus* 4.3. Stability prediction models at the desired temperatures of both isolates were obtained by calculating the *k* values at the desired temperatures with Arrhenius equations, as shown in Figure 8. The accelerated stability of the freeze-dried spores in Figure 9 showed that the predicted accelerated stability of *L. fusiformis* 5.1 and *L. xylanilyticus* 4.3 were highly accurate, with no significant differences from the experimental value at the storage temperatures of 8, 15 and 37 °C. The spore viability of both isolates was slightly changed after being stored at 8 and 15 °C and could be maintained up to 30 days. The spore viability of both isolates greatly decreased when incubated at 37 and 55 °C. From the equation (Figure 8), the predicted spore viabilities of *L. fusiformis* 5.1 and *L. xylanilyticus* 4.3 at ambient temperature in Thailand (28 ± 2 °C) for 30 days were 5.19 × 10^5^ and 2.56 × 10^6^ CFU/mL (23.92% and 20.81% remain), respectively.

## 4. Discussion

This study aimed to isolate high-yield urease-producing bacteria for MICP application. With a two-step screening process using Christensen’s urea agar and high buffered Stuart’s urea broth, four ureolytic isolates were obtained from soil in mangrove forests and sandy loam in Thailand. 16S rRNA analysis revealed that all isolates belonged to the genus *Lysinibacillus,* which is among well-known urease producing bacteria, and one to high alkalinity. It has been often used in several MICP studies of ureolysis activity [9,11,21,24,37]. Three isolates were obtained from soil collected from mangrove forests. Isolates 3.7 and 5.1 were identified as *Lysinibacillus fusiformis*, which has also been observed in calcareous soil [2]. However, the isolate 2.2 did not belong to any described species and is probably a novel species of the genus. One isolate, the isolate 4.3, which was identified as *Lysinibacillus xylanilyticus*, was obtained from a sandy loam sample collected from a factory site experiencing ground subsidence. Determinations of urease activity, EPS formation and CaCO_3_ precipitation activity under experimental conditions revealed that the most potentially useful isolate with the highest urease production and CaCO_3_ precipitation activities was *L. fusiformis* 5.1, followed by *L. xylanilyticus* 4.3. The urease activity was observed at 6 h after incubation and reached 4.40 × 10^3^ and 3.93 × 10^3^ unit/L, respectively, within 48 h. The CaCO_3_ precipitant in the cultures of *L. fusiformis* 5.1 and *L. xylanilyticus* 4.3 in the presence of 0.4 M CaCl_2_ was approximately 22.85–24.15 and 20.90–21.34 mg/mL, respectively. It is worth noting that even though *L. xylanilyticus* 4.3 exhibited great potential in terms of MICP capability in the laboratory experiment, its original habitat involved a problem of ground subsidence. Much research has reported that many MICP-inducing bacteria are naturally ubiquitous in soils and promote CaCO_3_ precipitation through various metabolic pathways such as ureolysis, ammonification, denitrification, methane oxidation and sulfate reduction [11,25,26,38,39]. However, in this case, it might occur because the environmental factors at the factory site were not appropriate for the MICP process, or due to a low population of MICP-inducing bacteria in the habitat. The first report of MICP activity of *L. xylanilyticus* was reported by [17]. The strain was locally isolated from a subarctic region (Hokkaido, Japan) and its urease was highly heat inactivated and not stable at 30 °C and above. On the other hand, *L. xylanilyticus* 4.3, which was isolated from a subtropical region, exhibited high MICP capability at the ambient temperature of Thailand. The average maximum temperature in Thailand can be 30 ± 3 °C in winter and up to 33 ± 3 °C in summer. It can be warmer in concrete and the surface of soil exposed under sunlight. Hence, heat-stable urease is preferable in tropical regions.

Most of the previous research in laboratory conditions observed CaCO_3_ precipitation by cultivating ureolytic bacteria in media containing 2% urea and calcium ions [1,2,24,36]. However, we investigated CaCO_3_ precipitation by separating this process into two steps. First, the ureolytic bacteria were grown and produced urease in urea broth without calcium ions to avoid the adverse impacts of calcium ions on bacteria growth and urease production [36]. During this process, carbonate ions were produced by urea hydrolysis and accumulated in the culture broth. Then, a CaCl_2_ solution was applied to the culture medium to induce CaCO_3_ precipitation. A large amount of CaCO_3_ particles were immediately formed, and the alkaline pH (>9.0) of the culture broth was decreased to approx. 8.0. The XRD patterns confirmed that the CaCO_3_ polymorph was calcite [38]. Hence, the method might be suitable for field applications such as biocementation, which requires large amounts of CaCO_3_ formation. The alkaline pH also instantly fell close to neutral, which had less impact on the environment in terms of pH. Nevertheless, the optimum condition for the CaCO_3_ precipitation of these two isolates requires further investigation to improve the MICP process in field application.

Bacterial cells and EPS serve as nucleation sites for extracellular CaCO_3_ precipitation, which is in agreement with previous studies. Environmental factors, such as aeration, temperature, pH, and specific proteins in EPS can affect crystalline morphology and formation of different CaCO_3_ polymorphs [2,21,23,39]. In the EPS formation assay (microplate scale), urea greatly inhibited EPS formation of all bacterial isolates. Only *L. xylanilyticus* 4.3 and *Lysinibacillus* sp. 2.2 were still able to produce EPS in the presence of urea. Therefore, *L. xylanilyticus* 4.3,which had both high urease activity and EPS formation, was selected to perform material crack filling, along with *L. fusiformis* 5.1, the highest urease producing isolate. Under SEM visualization, the smooth crystalline layer of *L. xylanilyticus* 4.3 was clearly observed in the cracks in the mortar specimens, forming a smooth surface of the calcite layer. The crystal structure was hardly visible, which might be due to the EPS formation of *L. xylanilyticus* 4.3. However, irregular spherical and rhombohedral crystals were found in *L. fusiformis* 5.1 (Figure 7). There are several reports of different CaCO_3_ polymorphs, aragonite, calcite and vaterite, produced by MICP-inducing bacteria [2,23]. However, the EDS analysis showed that only calcite was observed as the main polymorph in this study.

In order to successfully develop a commercial product, a shelf-life prediction of the MICP-inducing bacteria is necessary. Spore-forming bacteria have more advantages in this aspect because the bacterial spores survive longer than vegetative cells under unfavorable conditions [7,9]. In this study, accelerated storage testing was used for predicting the storage time limits of the spore viability of the ureolytic bacteria at various storage temperatures. The best storage temperature of *L. fusiformis* 5.1 and *L. xylanilyticus* 4.3 was a low temperature (8 °C). The predicted spore viability of *L. fusiformis* 5.1 after storage at 8 °C for 30 and 60 days was 1.59 × 10^6^ and 1.10 × 10^6^ CFU/mL, respectively (73.27% and 50.69% remain). In the case of *L. xylanilyticus* 4.3, the spore viability was estimated to be 4.83 × 10^6^ and 2.39 × 10^6^ CFU/mL (39.27% and 19.43% remain) after storage at 8 °C for 30 and 60 days, respectively. The specific rate of degradation per day (*k*) at the experimental temperature of *L. fusiformis* 5.1 was less than that of *L. xylanilyticus* 4.3, indicated by the fact that *L. fusiformis* 5.1 had higher spore viability than *L. xylanilyticus* 4.3. In this study, skimmed milk powder, which is a common protective carrier for bacterial preservation, was used. Other protective carriers should be further investigated to improve spore viability and germination. Studying spore viability is not only beneficial for the evaluation of product shelf-life, but also important in the development of the MICP technology for self-healing concrete. The bacterial spores are added and embedded inside the concrete during the mixing process. High alkalinity (pH 12–13), compressive strength and the shearing force within the concrete produce an extremely harsh environment for the bacteria. Hence, the survival and viability of the MICP-inducing bacteria, which are the core strategy for CaCO_3_ formation, are the crucial factors for self-healing technology [1,7,9,13,36]. Many researchers have reported that several protective carriers, such as diatomaceous earth [40], polyurethane, silica gel [41], hydrogel-based microcapsule [42], and alginate microcapsules [9,24], were able to enhance the bacterial survival rate and the MICP process.

## 5. Conclusions

This study reports newly isolated ureolytic bacteria in the genus *Lysinibacillus*, isolated from mangrove forest soil and sandy loam in Thailand. The highest urease production and CaCO_3_ formation were observed in *L. fusiformis* 5.1, followed by *L. xylanilyticus* 4.3. The main CaCO_3_ polymorph of both isolates was calcite. Crack filling in mortar specimens showed that *L. xylanilyticus* 4.3, an EPS producing isolate, formed a smooth layer of calcite, whereas *L. fusiformis* 5.1, which was not able to produce EPS, precipitated irregular spherical and rhombohedral calcite crystals. These two isolates have robust characteristics of potential ureolytic bacteria suitable for MICP application.

## Figures and Tables

**Table 1:** Identification results based on the 16S rRNA gene sequence similarity and the positive results of Stuart’s urea broth.

Identification Result	Closest Species (%Similarity)	Source	pH Change in Stuart’s Urea Broth	Final pH
*Lysinibacillus* sp. 2.2	*Lysinibacillus fusiformis* NBRC 15717^T^ (98.35%)	Phra Chedi Klang Nam Mangrove Forest Learning Center, Rayong	+++	9.86
*Lysinibacillus fusiformis* 3.7	*Lysinibacillus fusiformis* NBRC 15717^T^ (99.32%)	Phra Chedi Klang Nam Mangrove Forest Learning Center, Rayong	+++	10.00
*Lysinibacillus xylanilyticus* 4.3	*Lysinibacillusxylanilyticus* DSM 23493^T^ (99.26%)	Bowin, Si Racha district, Chon Buri	+	9.83
*Lysinibacillus fusiformis* 5.1	*Lysinibacillus fusiformis* NBRC 15717^T^ (99.42%)	Black Sand Beach and Mangrove Forest Natural Tourism Center, Trat	+++	10.23

+++ Positive result within 24 h; ++ positive result within 36 h; + positive result within 48–72 h.

## Data Availability

All supporting data was submitted as supplementary materials.

## Supplementary Materials

The following supporting information can be downloaded at: https://www.mdpi.com/article/10.3390/microorganisms10050963/s1, Table S1: SD value of growth of bacterial isolates in NB, NB supplemented with 2% urea, TSB, and TSB supplemented with 2% urea; Table S2: SD value of urease activity of bacterial isolates grown on TSB supplemented with 2% urea at 150 rpm, 28 °C for 48 h; Table S3: SD value of experimental viability of bacterial spores in the accelerated storage test.
