# Supplementary material for: Biocalcifying Potential of Ureolytic Bacteria Isolated from Soil for Biocementation and Material Crack Repair
Source: Microorganisms. 2022 May 3;10(5):963. doi: 10.3390/microorganisms10050963 (PMC9143465; doi:10.3390/microorganisms10050963)
Supplement: Supplementary file 1 [file microorganisms-10-00963-s001.zip › microorganisms-1694346-supplementary.pdf]

Supplementary Table S1. SD value of growth of bacterial isolates in NB, NB supplemented with 2% urea, TSB, and TSB supplemented with 2% urea.

Time (h)	OD_{600}															
	NB				NB supplemented with 2% urea				TSB				TSB supplemented with 2% urea			
	Isolate 2.2	Isolate 3.7	Isolate 4.3	Isolate 5.1	Isolate 2.2	Isolate 3.7	Isolate 4.3	Isolate 5.1	Isolate 2.2	Isolate 3.7	Isolate 4.3	Isolate 5.1	Isolate 2.2	Isolate 3.7	Isolate 4.3	Isolate 5.1
0	0.002	0.001	0.001	0.001	0.001	0.008	0.001	0.000	0.001	0.000	0.000	0.001	0.002	0.001	0.000	0.003
2	0.001	0.001	0.000	0.003	0.000	0.001	0.002	0.001	0.002	0.004	0.001	0.003	0.001	0.001	0.000	0.000
4	0.002	0.003	0.002	0.004	0.002	0.004	0.015	0.001	0.007	0.003	0.005	0.005	0.011	0.014	0.014	0.001
6	0.026	0.003	0.006	0.004	0.003	0.002	0.002	0.003	0.005	0.003	0.002	0.007	0.028	0.014	0.043	0.013
8	0.002	0.003	0.004	0.005	0.003	0.006	0.009	0.017	0.002	0.012	0.012	0.003	0.014	0.003	0.054	0.008
10	0.001	0.001	0.003	0.006	0.005	0.003	0.004	0.003	0.011	0.008	0.005	0.001	0.021	0.001	0.028	0.025
12	0.002	0.004	0.005	0.001	0.006	0.003	0.004	0.005	0.009	0.011	0.011	0.005	0.004	0.022	0.016	0.019
18	0.000	0.002	0.004	0.003	0.008	0.003	0.005	0.004	0.007	0.005	0.006	0.000	0.006	0.002	0.009	0.004
24	0.006	0.006	0.012	0.003	0.003	0.004	0.001	0.000	0.042	0.009	0.005	0.008	0.005	0.007	0.042	0.006
30	0.004	0.002	0.004	0.001	0.003	0.008	0.004	0.003	0.009	0.003	0.010	0.012	0.003	0.006	0.01	0.015
36	0.002	0.003	0.002	0.011	0.005	0.001	0.007	0.001	0.012	0.013	0.009	0.012	0.005	0.006	0.014	0.004
42	0.002	0.014	0.006	0.004	0.002	0.004	0.002	0.002	0.007	0.017	0.006	0.003	0.004	0.004	0.004	0.003
48	0.003	0.001	0.003	0.003	0.003	0.006	0.005	0.002	0.009	0.013	0.008	0.005	0.008	0.03	0.006	0.004

Supplementary Table S2. SD value of urease activity of bacterial isolates grown on TSB supplemented with 2% urea at 150 rpm, 28 °C for 48 h.

**Time (h)**	**Urease activity (unit/L)**			
	**Isolate 2.2**	**Isolate 3.7**	**Isolate 4.3**	**Isolate 5.1**
0	0.001	0.001	0.001	0.001
8	0.022	0.007	0.004	0.008
12	0.003	0.020	0.017	0.016
24	0.001	0.009	0.004	0.013
48	0.016	0.031	0.016	0.003

Supplementary Table S3. SD value of experimental viability of bacterial spores in the accelerated storage test.

**Time (days)**	***L. fusiformis* 5.1**				***L. xylanilyticus* 4.3**			
	**8 °C**	**15 °C**	**37 °C**	**55 °C**	**8 °C**	**15 °C**	**37 °C**	**55 °C**
0	0.0181	0.0181	0.0181	0.0181	0.0011	0.0011	0.0011	0.0011
7	0.0096	0.0499	0.0108	0.0677	0.0254	0.0451	0.0791	0.0264
14	0.0189	0.0708	0.0504	0.0097	0.0162	0.0289	0.0272	0.0231
21	0.0123	0.0222	0.0107	0.0802	0.0341	0.1105	0.0881	0.0696
30	0.0179	0.0286	0.0031	0.0136	0.0063	0.0231	0.0584	0.0356
